# Recombinant Expression and* In Vitro* Characterisation of Active Huwentoxin-IV

**DOI:** 10.1371/journal.pone.0083202

**Published:** 2013-12-06

**Authors:** Isabelle Sermadiras, Jefferson Revell, John E. Linley, Alan Sandercock, Peter Ravn

**Affiliations:** 1 Antibody Discovery and Protein Engineering, Research, MedImmune, Cambridge, United Kingdom; 2 Neuroscience *in vitro* Biology, Research, MedImmune, Cambridge, United Kingdom; Moffitt Cancer Center, United States of America

## Abstract

Huwentoxin-IV (HwTx-IV) is a 35-residue neurotoxin peptide with potential application as a novel analgesic. It is a member of the inhibitory cystine knot (ICK) peptide family, characterised by a compact globular structure maintained by three intramolecular disulfide bonds. Here we describe a novel strategy for producing non-tagged, fully folded ICK-toxin in a bacterial system. HwTx-IV was expressed as a cleavable fusion to small ubiquitin-related modifier (SUMO) in the cytoplasm of the SHuffle T7 Express *lysY Escherichia coli* strain, which allows cytosolic disulfide bond formation. Purification by IMAC with selective elution of monomeric SUMO fusion followed by proteolytic cleavage and polishing chromatographic steps yielded pure homogeneous toxin. Recombinant HwTx-IV is produced with a C-terminal acid, whereas the native peptide is C-terminally amidated. HwTx-IV(acid) inhibited Na_v_1.7 in a dose dependent manner (IC_50_ = 463-727 nM). In comparison to HwTx-IV(amide) (IC_50_ = 11 ± 3 nM), the carboxylate was ~50 fold less potent on Na_v_1.7, which highlights the impact of the C-terminus. As the amide bond of an additional amino acid may mimic the carboxamide, we expressed the glycine-extended analogue HwTx-IV^G36^(acid) in the SUMO/SHuffle system. The peptide was approximately three fold more potent on Na_v_1.7 in comparison to HwTx-IV(acid) (IC_50_ = 190 nM). In conclusion, we have established a novel system for expression and purification of fully folded and active HwTx-IV(acid) in bacteria, which could be applicable to other structurally complex and cysteine rich peptides. Furthermore, we discovered that glycine extension of HwTx-IV(acid) restores some of the potency of the native carboxamide. This finding may also apply to other C-terminally amidated peptides produced recombinantly.

## Introduction

**Figure 1:**
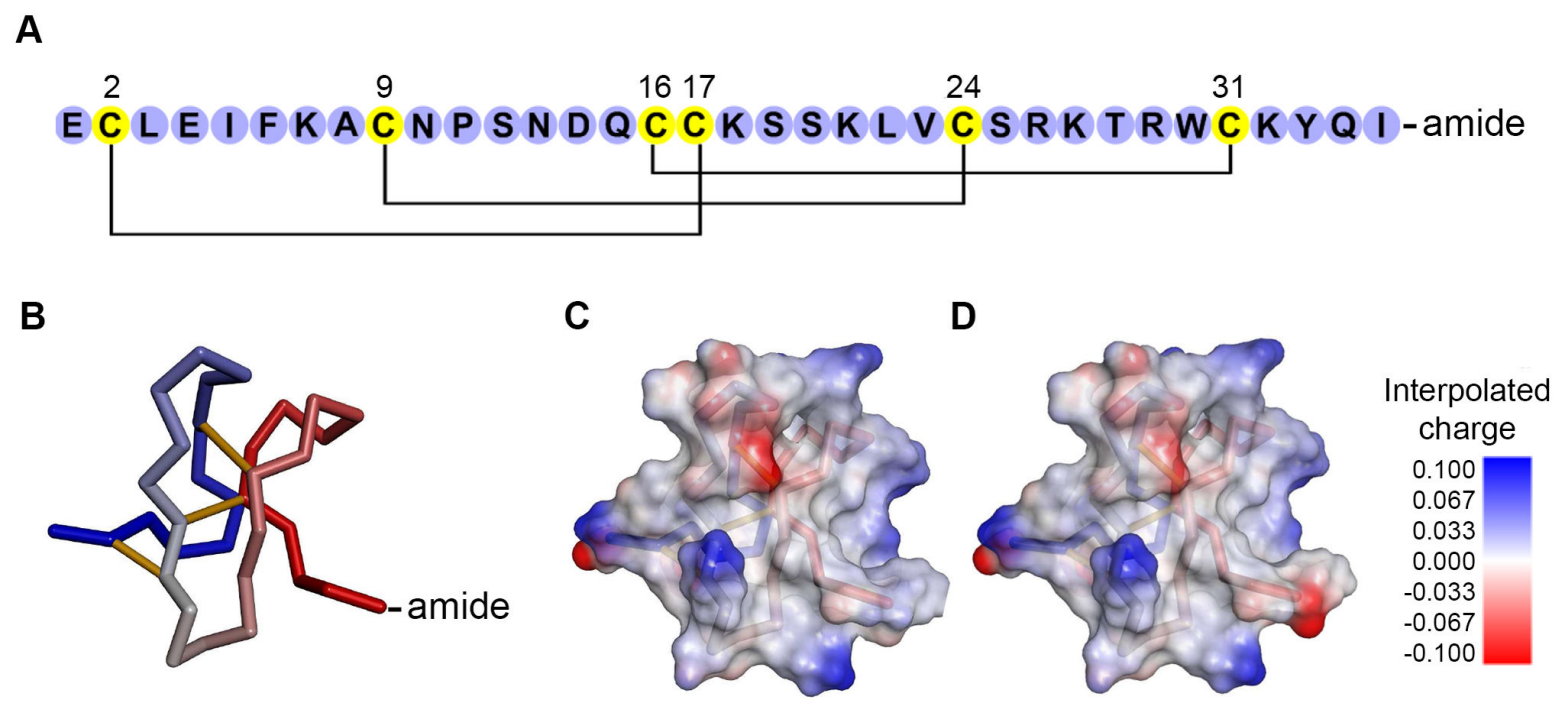
Representation of HwTx-IV. A) Amino acid sequence of native HwTx-IV (C-terminal amide) with the correct disulfide pairing. B) Structure of HwTx-IV (1mb6). The three disulfide bonds are shown in yellow. C-D) Interpolated charge solvent surfaces of HwTx-IV(amide) (C) and HwTx-IV(acid) (D) shows an important charge difference in the C-terminal region (Discovery Studio 3.5, Accelrys).

Huwentoxin-IV (HwTx-IV), also known as mu-theraphotoxin-Hh2a, is a 35-residue peptide toxin from the NaSpTx Family 1, originally isolated from the venom of the Chinese Bird Spider *Ornithoctonus huwena* [1,2]. HwTx-IV preferentially inhibits the neuronal voltage-gated sodium channels Na_v_1.2, Na_v_ 1.3 and Na_v_1.7 [3]. It is most potent at Na_v_1.7, which plays a crucial role in pain transduction with familial gain of function mutations in man being linked to several chronic pain disorders [4], while loss of Na_v_1.7 function results in congenital insensitivity to pain [5]. HwTx-IV belongs to the Inhibitor Cystine Knot (ICK) peptide family, characterised by a rigid and stable scaffold where three β-sheets are held together by three highly conserved disulfide bonds (Figure 1) [1,6]. The specificity and selectivity of HwTx-IV at Na_v_1.7 (>5 fold higher potency over Na_v_1.2 and Na_v_1.3) and the relative lack of efficacy at the cardiac Na_v_ isoform Na_v_1.5 [3] make HwTx-IV a promising starting point for the development of an analgesic drug targeting chronic pain. 

Small amounts of native HwTx-IV can be isolated from venom for scientific studies [1], however, in order to evaluate its therapeutic potential, alternative and more abundant sources are required. Fully folded and active HwTx-IV has been produced by solid-phase peptide synthesis and *in vitro* refolding [7]. We have used this strategy to generate HwTx-IV analogues for a structure-activity relationship based optimisation of the potency on Na_v_1.7 [8]. A recombinant expression system that produces correctly folded HwTx-IV would be a valuable alternative to synthesis and *in vitro* re-folding.

Despite their complicated tertiary structure, which is a challenge for recombinant expression, production of several ICKs has been reported, using both prokaryotic and eukaryotic systems [9–18]. ICKs have been expressed with cleavable fusion partners to increase solubility and/or to simplify purification (MBP [12], GST [14,15], His6-tag [10–13,17]). After purification, fusion partners are removed, using proteases [10–16] or chemical cleavage [16]. Alternatively, an auto-cleavable intein fusion tag can simplify the removal of the fusion partner [9]. A drawback of the fusion approach is that some amino acids may be left attached to the ICK after tag removal, depending on the cleavage system used [13,14].

As disulfide bonds cannot form efficiently in the reducing environment of *E. coli* cytoplasm, *in vitro* oxidative refolding of the linear reduced peptide may be required to obtain the native disulfide pairing [12–14]. Alternatively, the ICK-fusion protein can be directed to the periplasm where the oxidative environment allows for disulfide bond formation [10]. Successful ICK secretion using fusion to barnase or a flagellar protein has been reported [13,16]. However, disulfide bond formation in the periplasm is catalysed by the disulfide bond isomerase DsbA, which oxidises cysteine residues consecutively as they enter the periplasm [19,20], so this strategy could lead to mispairing and may not be suitable for all ICKs. Overexpression of the periplasmic disulfide bond isomerase DsbC may enhance the amount of correctly folded protein [21,22], and has been used successfully as a fusion partner for other disulfide-rich proteins, including HwTx-I [10,23]. Structurally, HwTx-IV is very similar to HwTx-I, but whereas HwTx-I seems to fold readily in several recombinant systems [9,10,14,17], HwTx-IV does not (unpublished findings). *E. coli* strains engineered for cytoplasmic expression and folding of disulfide-rich proteins, like Origami B and SHuffle, are another alternative to express folded ICKs [11,23–26]. 

Here, we investigated the expression of HwTx-IV in the *E. coli* strain SHuffle T7 Express *lysY* [26]. In addition to an oxidising cytosolic environment identical to Origami B (*Δgor ΔtrxB*), SHuffle expresses DsbC cytoplasmically [23,25,26]. We have fused HwTx-IV to the small ubiquitin-related modifier (SUMO) [27–30]. SUMO is reported to increase the solubility and expression yield of fusion proteins, and SUMO protease mediated cleavage is highly specific and does not leave residual amino acids at the N-terminus of the fusion partner. We have fine-tuned the IMAC purification to capture monomeric His6-SUMO-HwTx-IV(acid) from the soluble cell lysate. Full characterisation of HwTx-IV(acid) was performed after SUMO protease cleavage and polishing. We compared recombinant and synthetic HwTx-IV(acid), as well as the native toxin HwTx-IV(amide). An analogue with C-terminal glycine extension was produced in the SUMO/SHuffle system to mimic the native C-terminal amide, and also as a possible substrate for *in vitro* enzymatic amidation using Peptidylglycine α-amidating monooxygenase (PAM) [31–34]. 

## Materials and Methods

### Materials

The DH5α Z competent cells used for plasmid propagation were purchased from Zymo Research (Irvine, CA, USA). The expression strain SHuffle T7 Express *lysY* was purchased from New England Biolabs (Ipswich, MA, USA). All bacterial expression experiments were conducted using in house 2xTY media supplemented with 1% glucose and 30ug/ml Kanamycin. SDS PAGE gels were run using NuPAGE products from Invitrogen: NuPAGE 10% or 12% 1mm gels and MES running buffer. Gel staining was performed using Simplyblue SafeStain (Invitrogen) according to the supplier’s instructions. PAM was supplied by OriGene (Rockville, MD, USA). Chemicals for buffer preparations were ordered from Sigma Aldrich.

### Recombinant plasmid construction

A synthetic gene of HwTx-IV was constructed by Taq polymerase extension of two oligos, hwtx-for (GAGTGCTTAGAGATTTTTAAGGCATGCAACCCTTCAAATGACCAGTGCTGCAAGAGCTCGAAATTAGTTTGCAGTCGCAAAACCCGTTGGTGTAAATACCAAATT) and hwtx-back (TTATTATCAAATTTGGTATTTACACCAACGGG) and cloned into pET-SUMO (Invitrogen) by TA cloning as instructed by the manufacturer. Sequencing confirmed the correct construct for direct fusion of SUMO and HwTx-IV followed by three stop codons. The construct for the production of HwTx-IV^G36^ was generated using another reverse oligo, HwTx-IV-Gly-back (TTATTATCATCCAATTTGGTATTTACACCAACGGG).

### Expression

The expression vector was transformed into the *E. coli* SHuffle T7 Express *lysY* strain using the supplier’s protocol. A single colony from a fresh plate was used to inoculate a primary culture, which was grown overnight at 30°C and 280rpm. The following morning, a larger culture was inoculated at OD_600nm_= 0.05 using the overnight pre-culture. The flask was incubated at 30°C and 280rpm until OD_600nm_ reached 0.6-0.8. IPTG was then added to a final concentration of 0.5mM to induce expression of the His6-SUMO-HwTx-IV(acid) fusion. The culture was immediately transferred to a 16°C incubator for overnight expression. The bacteria were harvested by centrifugation (8,000g, 15 minutes, 4°C). The pellet was stored at -20°C until purification.

### Cell lysis and soluble protein extraction

The bacteria pellet was lysed using BugBuster Master Mix (Novagen), which contains rLysozyme and Benzonase, facilitating rapid cell lysis, DNA content reduction and efficient soluble protein extraction. 12.5ml BugBuster Master Mix, supplemented with protease inhibitors (Complete, EDTA-free, Roche), was used per 100ml culture pellet. After 30 minutes agitation at room temperature, the lysate was spun down (17,000g, 15 minutes, 4°C) and filtered (0.2µM). To lower unspecific binding to the resin, imidazole was added to the lysate (final concentration 50mM) using a 2M stock solution.

### Capture IMAC

The cleared lysate was purified on a 5ml HiTrap Chelating column (GE Healthcare) charged with NiSO_4_. The purification was automated using an ÄKTA Purifier system (GE Healthcare). Two buffers (Buffer A: 10mM Tris/HCl pH 8.0, 500mM NaCl and Buffer B: 10mM Tris/HCl pH 8.0, 500mM Imidazole, 500mM NaCl) were used to generate the different concentrations of imidazole and the imidazole gradient. The flow rate was maintained at 5ml/min. The column was equilibrated for 5 column volumes (CV) at 50mM imidazole (10% buffer B). The lysate was then loaded (typically 150ml), followed by a 50mM imidazole wash step (60 CV). Elution was performed using an imidazole gradient (6.4 CV, 50-250mM imidazole) to understand the elution pattern of His6-SUMO-HwTx-IV(acid). A two-step elution profile was developed using this information (20 CV at 105mM imidazole, and 20 CV at 500mM imidazole). 5ml fractions were collected and analysed by SDS PAGE.

### Concentration and buffer exchange

Once combined, the desired fractions were concentrated and buffer exchanged using Amicon Ultra-15 centrifugal filter units (3kDa molecular weight cut-off, Millipore). They were first loaded onto as many Amicon devices as necessary (15ml sample each), and spun at room temperature for 20 minutes at 4,000g. The filtrate was discarded and 12ml fresh cleavage buffer (20mM Tris/HCl pH8.0, 150mM NaCl) was added to the reservoir and mixed to the concentrated sample. The devices were then spun down for 20 minutes at 4,000g again. This buffer exchange step was repeated twice. A final concentration step was then performed by spinning the devices for another 20 minutes at 4,000g. The concentrate volume was approximately 1ml in each Amicon device. The concentrates were combined and spun to remove any precipitate (17,000g, 5 minutes, 4°C). 

### SUMO protease cleavage

SUMO protease (Tebu-bio) was added to the concentrated fusion protein to a final concentration of 20U/mg. The cleavage reaction was incubated at 30°C and 250rpm overnight. Tebu-bio SUMO protease was used for the cleavage because it is supplied in a detergent-free buffer, which does not interfere with the electrophysiology screening. The supplier recommends using 1-2mM DTT for optimal SUMO protease activity, however, such concentrations of DTT would reduce His6-SUMO-HwTx-IV(acid). We decided to use SUMO protease with a low concentration of reducing agent (0.1mM DTT) to avoid reduction of the disulfide bonds.

### Depletion IMAC

The following morning, the cleavage reaction was spun (17,000g, 5 minutes, 4°C) before loading onto the gravity IMAC column (packed in house with 700ul resin Ni-NTA resin). The column was first equilibrated with 5ml of dep-IMAC buffer (10mM Tris pH 8.0, 10mM imidazole, 300mM NaCl). The cleavage reaction was then loaded onto the column. The column was washed with 4ml of dep-IMAC buffer. During the loading and washing steps, 700ul fractions were collected and analysed by SDS PAGE. The positive fractions were then combined. The amount of cleaved peptide produced was determined by absorbance at 280nm.

### Preparative Reverse Phase HPLC

The pooled depletion-IMAC fractions were polished using Reversed Phase HPLC on a Varian Prep-Star SD-1 system. The peptide was diluted in a solution of water:Acetonitrile:TFA 1:1:1 (5% v/v) and applied directly to the stationary phase (Agilent Polaris C8-A, 250x10mm 5μm). A gradient from 10 to 50% acetonitrile (0.1% TFA v/v) in 30 minutes at ambient temperature was used. The appropriate fractions were pooled and lyophilised.

### LC-MS and analytical RP-HPLC

For LC-MS analysis, 10ul of material was loaded onto a Waters X-Bridge C18 stationary phase (4.6 x 100 mm, 3μm). A 10 minutes gradient from 10% to 90% acetonitrile (0.1% TFA v/v) at ambient temperature was used to resolve the analytes. The eluted compounds were detected by UV absorption at 210nm and ionisation using a Waters 3100 mass detector (ESI^+^ mode). Molecular masses were compared to theoretical values calculated for the reduced and oxidised peptide.

Analytical RP-HPLC was performed using a Varian 920-LC system using an Agilent Polaris C8-A column (4.6x100mm 3μm). 50ul was loaded and a gradient from 10 to 50% acetonitrile (0.1% TFA v/v) in 15 or 30 minutes at 40°C. Analytes were detected by UV absorbance at 210nm.

### MALDI-MS analysis

Peptide samples were prepared for mass spectrometry by addition of TFA to a final concentration of 0.1%, then bound to Zip-tip C18 tips (Millipore), washed with 0.1% TFA and eluted into MALDI matrix solution (10mg/ml alpha-cyano-4-hydroxycinnamic acid in 50:50:0.1 acetonitrile:water:TFA). 1µL spots were applied to a MALDI target and air-dried. Mass spectra were acquired on an AB4800 ToF/ToF mass spectrometer in positive reflector mode, with external calibration on a peptide calibrant mixture. Theoretical isotope distributions were calculated from molecular formula using the instrument’s analysis software.

### Electrophysiology using Sophion QPatch^TM^

hNa_v_1.7 alpha HEK 293 cell line (NM_002977.2) was obtained from Millipore and cells were cultured in a 50/50 mix of DMEM and Hams F-12 growth media supplemented with 10% FBS. Cells were passaged by Detachin (Genlantis) treatment (5 minutes), seeded 48-72 hours prior to experiments and used between passage 5 and 10. Cells were harvested at 70% confluency and resuspended by trituration in 8ml of Ex-cell ACF CHO serum free media (Sigma) + 20mM HEPES giving a cell density of 2-3x10^6^cells/ml.

Solutions for electrophysiology: Extracellular solution contained (in mM) NaCl (145), KCl (2), CaCl_2_ (2), MgCl_2_ (1), HEPES (10), pH 7.4 with NaOH, osmolarity adjusted to 300 mOsm with sucrose. Intracellular solution contained (in mM), CsF (140), NaCl (10), EGTA (1), HEPES (10), pH 7.2 with CsOH, osmolarity adjusted to 325 mOsm with sucrose. 

HwTx-IV stocks (1mM) were made up by reconstituting lyophilised protein in extracellular buffer. Serial dilutions were made in extracellular buffer + 0.1% BSA. Cumulative dose response curves were constructed with doses of 1e-10 to 1e-5M (100pM to 10µM). Each dose was applied for 10 minutes with 3x5µl compound additions spaced 2 seconds apart (Note that complete solution exchange is achieved with volumes of ~2µl). Five doses were tested on each cell with no appreciable rundown of current measured in time matched control experiments.

Na_v_1.7 currents were measured using the automated electrophysiology platform Qpatch 16X (Sophion) in single hole configuration. Mean series resistance was < 6MΩ and seal resistance was > 1GΩ for the entirety of the experiment. To measure Na_v_1.7 currents, membrane potential was held at -80 mV then stepped to -120mV for 100ms to remove fast inactivation, followed by a step to 0mV for 10ms to activate Na_v_1.7 then returned to -120mV for 100ms. This voltage protocol was repeated at a rate of 0.2Hz with a the membrane potential held at -80mV between sweeps. Data were sampled at 10kHz and filtered using a 4 pole Bessel filter at 3kHz. P/N leak subtraction was utilized with a leak sweep of -10%. Series resistance was compensated 75% as standard. Liquid junction potentials were not corrected.

Peak inward current at 0mV was normalised to cell capacitance (pA/pF). Steady state current density in response to each drug concentration was normalised to vehicle control (extracellular solution + 0.1% BSA) and plotted as fractional current. Data were fit with a variable slope dose response curve in graphpad Prism using the equation Y=Bottom + (Top-Bottom)/(1+10^((LogIC50-X)*HillSlope)) constrained so that Bottom must be 0.

### *In vitro* amidation of HwTx-IV^G36^(acid)

3500pmol of HwTx-IV^G36^(acid) was incubated for 24 hours at 37°C with 2ug of PAM in amidation buffer (50mM MES pH6.0, 5mM Ascorbic acid, 1µM copper sulfate, 0.01% Catalase). The reaction was frozen until MALDI-MS analysis.

## Results

### Recombinant expression of His6-SUMO-HwTx-IV(acid)

His6-SUMO-HwTx-IV(acid) was expressed in SHuffle T7 Express *lysY*. Initially, two expression conditions were compared: 4 hours at 30°C and overnight at 16°C (data not shown). Soluble fusion protein accumulation was observed at both temperatures. SDS-PAGE analysis of the IMAC purified His6-SUMO-HwTx-IV(acid) revealed a mixture of monomers and covalent multimers of fusion protein, suggesting that SHuffle T7 Express *lysY* cells are efficient for cytoplasmic disulfide bond formation (data not shown). However, the cytoplasmic expression of DsbC in SHuffle did not ensure exclusive production of the correctly folded monomers, as some multimers with intermolecular disulfide bridges were co-expressed. A larger amount of insoluble and multimeric fusion protein was produced after 4 hours at 30°C. To maximise production of soluble and correctly folded protein, we chose to express His6-SUMO-HwTx-IV(acid) at 16°C overnight.

### Selective elution of monomeric His6-SUMO-HwTx-IV(acid) using IMAC

**Figure 2:**
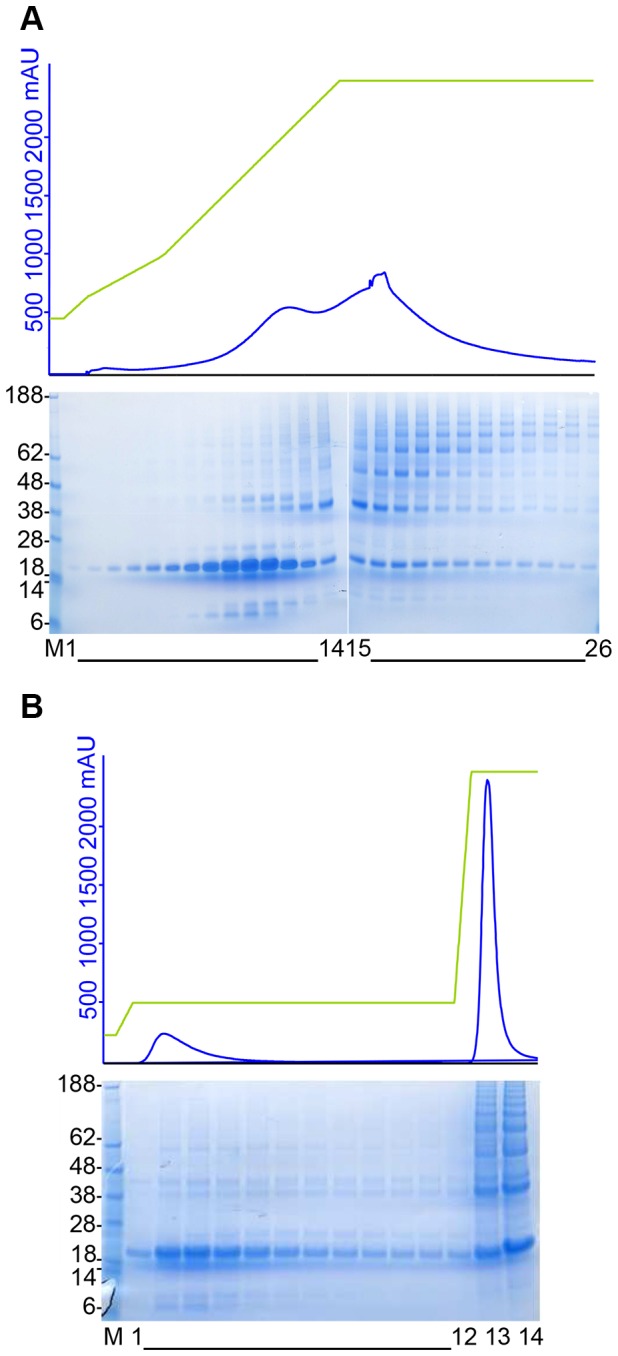
IMAC purification of His6-HwTx-IV(acid). A) Slow gradient elution IMAC. Lane M, Seeblue pre-stained molecular weight marker (Invitrogen); Lanes 1 to 26, fractions collected during imidazole gradient elution. Elution of monomers starts at 60mM imidazole. At 105mM imidazole, only monomers elute. B) Capture IMAC with step elution at 105mM Imidazole. Lane M, Seeblue pre-stained molecular weight marker (Invitrogen); Lanes 1 to 12, fractions collected during the 105mM imidazole elution; Lanes 13 and 14, fractions collected during the second elution step at 500mM imidazole.

Standard IMAC purification of His6-SUMO-HwTx-IV(acid) captures both monomeric and multimeric species. However, as multimers have several His6-tags per molecule, they bind more tightly to the IMAC resin than monomers with a single His6-tag. Consequently, monomers should elute more readily. A slow gradient elution with imidazole was run and demonstrated this effect (Figure 2, A). A concentration of imidazole of 105mM led to selective elution of the monomers, while the multimers remained bound to the resin. An elution protocol using a 105mM imidazole step was successfully used to elute the monomers in a single step purification (Figure 2, B). The few protein contaminants that co-eluted will be removed completely during the depletion IMAC (see below). A second step at a high imidazole concentration was used to evaluate the compounds which remained bound to the resin. It showed that a fraction of the monomers did not elute during the first elution step (Figure 2, B). A higher concentration of imidazole during the first elution step could therefore increase the overall yield of monomers, but could also lead to the elution of higher order multimers.

### Cleavage of fusion protein and polishing of HwTx-IV(acid)

**Figure 3:**
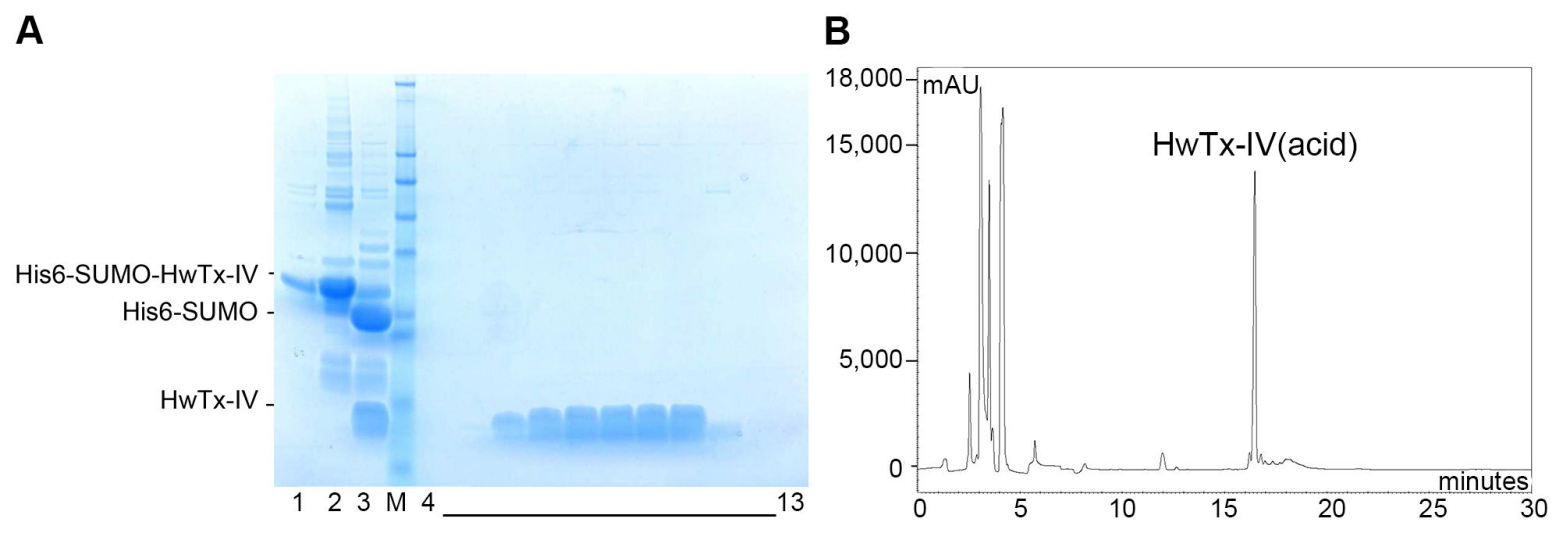
Cleavage and purification of HwTx-IV(acid). A) SDS-PAGE analysis of the cleavage reaction and depletion IMAC. Lane 1, Combined capture IMAC fractions; lane 2, Concentrated IMAC fractions in cleavage buffer; lane 3, Concentrated IMAC fractions after overnight SUMO protease cleavage; lane M, Seeblue protein MW marker; lanes 4 to 13, Depletion IMAC fractions. B) Polishing by preparative RP-HPLC. Chromatogram of the RP-HPLC polishing step showing that one main peak is observed. The corresponding fractions were combined and lyophilised.

The IMAC purified His6-SUMO-HwTx-IV(acid) was concentrated and digested with SUMO protease resulting in two main products, free HwTx-IV(acid) and His6-SUMO, as shown by SDS-PAGE (Figure 3, A). The cleavage reaction was efficient despite the sub-optimal conditions (low DTT concentration), and only a minor fraction of the fusion protein remained uncleaved. With the exception of HwTx-IV(acid), the components in the cleavage reaction all contain a His6-tag. As a result, they were depleted efficiently using IMAC, while recovering the pure HwTx-IV(acid) in the flow through (Figure 3, A). An additional benefit of the depletion IMAC is that the bacterial proteins that co-eluted with His6-SUMO-HwTx-IV(acid) during the capture IMAC, also bind to the depletion IMAC resin, resulting in a very high purity of HwTx-IV(acid) in the flow through. As a final polishing step, the free toxin was purified by preparative RP-HPLC (Figure 3, B).

### Characterisation of purified HwTx-IV(acid)

**Figure 4:**
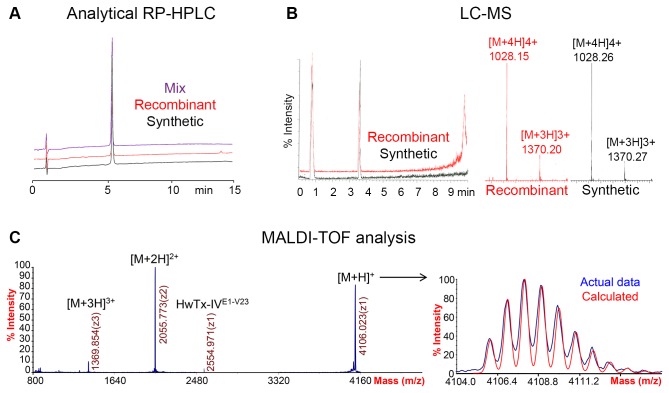
Characterisation of recombinant HwTx-IV(acid). A) Analytical RP-HPLC. The recombinant and synthetic HwTx-IV(acid) were analysed separately and combined (1:1). B) LC-MS analysis of the recombinant and synthetic HwTx-IV(acid). The peptides are identical. C) MALDI-TOF analysis of recombinant HwTx-IV(acid). The calculated and observed isotope distributions and m/z ions match. A minor cleaved form was detected: HwTx-IV^E1-V23^. The theoretical m/z ions are: [M+H]^+^ = 4105.928 m/z, [M+2H]^2+^ = 2053.464 m/z, [M+3H]^3+^ = 1369.981 m/z and [M+4H]^4+^ = 1027.232 m/z.

The recombinant HwTx-IV(acid) was compared to correctly folded synthetic HwTx-IV(acid) prepared in-house [8]. The analytical reversed phase HPLC traces of the peptides were identical, as well as the trace for a mixture of the two toxins, indicating that the two compounds have identical conformation (Figure 4, A). Analytical LC-MS of the recombinant HwTx-IV(acid) gave rise to a single peak on the LC trace at the same retention time as the peak of synthetic HwTx-IV(acid). The corresponding mass spectrometry analysis of the two peptides also gave identical peaks corresponding to the calculated m/z for oxidised HwTx-IV(acid) (Figure 4, B). The recombinant and synthetic HwTx-IV(acid) have identical properties and purity levels according to LC-MS and RP-HPLC analysis. The SUMO/SHuffle mediated *in vivo* folding is therefore as efficient as *in vitro* refolding.

MALDI Mass Spectrometry analysis of recombinant HwTx-IV(acid) showed a [M+H]^+^ ion with a monoisotopic mass of 4106.023 *m/z*, consistent with the expected mass for the peptide containing three disulfide-bonds: 4105.928 *m/z* (Figure 4, C). The multiple charged ions [M+2H]^2+^ and [M+3H]^3+^ also matched the theoretical m/z. The observed isotope distribution is identical to the calculated distribution from the molecular formula of the fully oxidised peptide, indicating that partially-reduced forms are absent or negligible components. Finally, MALDI MS analysis revealed a cleaved form of HwTx-IV(acid) with two disulfides (HwTx-IV^E1-V23^), which was not detected by LC-MS or RP-HPLC. This form must have an incorrectly formed disulfide bridge, since HwTx-IV^E1-V23^ contains only one native bond (Figure 1). As MALDI MS is a very sensitive technique but cannot be used quantitatively, it is likely that this cleaved form is present at very low concentration, since it was not detected by the quantitative methods. In conclusion, the recombinant HwTx-IV(acid) is of very high purity and in the same oxidation state than synthetic HwTx-IV(acid).

### The electrophysiological properties of HwTx-IV(acid)

**Figure 5:**
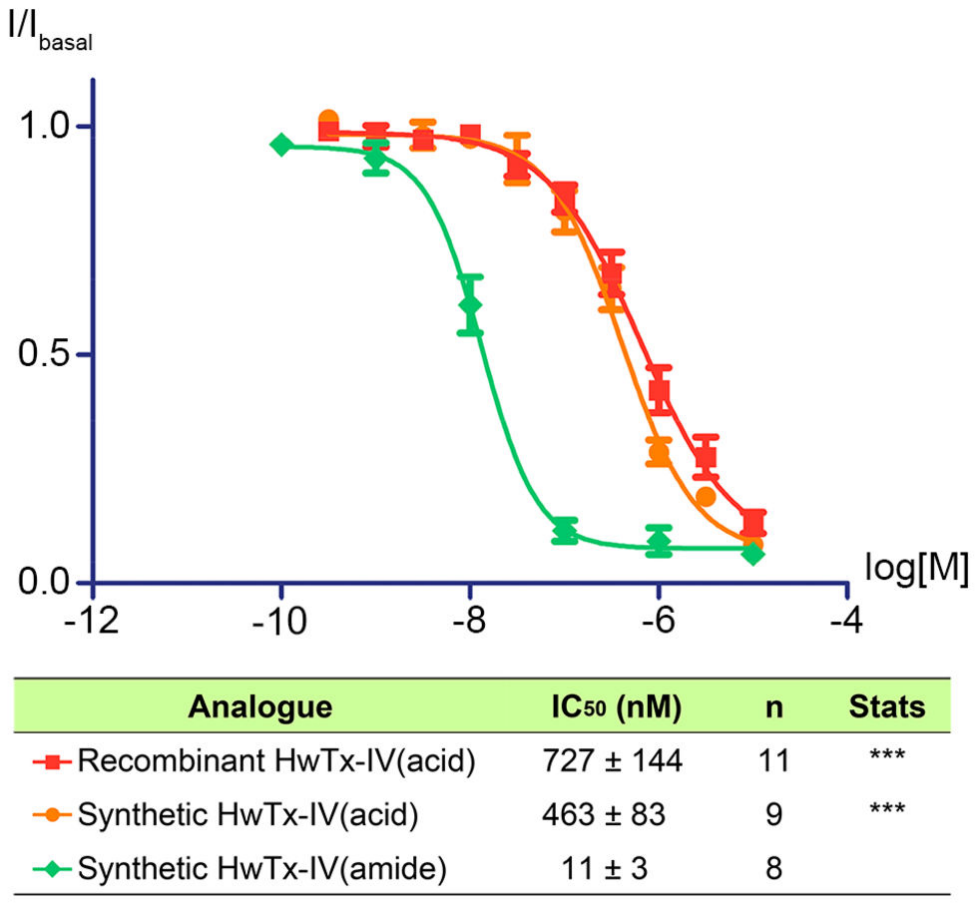
Electrophysiological characterisation of HwTx-IV(acid/amide) potency at hNa_v_1.7. Na_v_1.7 currents were measured from HEK cells stably expressing the hNa_v_1.7 alpha subunit using whole cell voltage clamp. Peak sodium current in the presence of increasing doses of HwTx-IV (10 minutes incubation per dose) is plotted as a fraction of basal current (I/Ibasal). Statistics indicate significant difference in IC_50_ between selected groups and synthetic HwTx-IV(amide), (one-way ANOVA with Tukey post-test; p<0.001, ***). No significant difference was observed between the recombinant and synthetic HwTx-IV(acid).

Both synthetic and recombinant HwTx-IV(acid) were analysed using an automated patch clamp system (Qpatch), on a stable cell line expressing the *h*Na_v_1.7 alpha subunit (Figures S1 and S2). As shown in Figure 5, they were equally potent on Na_v_1.7 with IC_50_ values of 727 ± 144 nM and 463 ± 83 nM, respectively (n = 9-11). These IC_50_ values are not significantly different, and confirm that both peptides are correctly folded. In comparison, synthetic HwTx-IV(amide) was markedly more potent with an IC_50_ of 11 ± 3 nM, which is in good agreement with the previously reported potency [1]. The potency difference between recombinantly produced HwTx-IV(acid) and synthetic HwTx-IV(amide) demonstrates the importance of the C-terminus. The charge at the C-terminus varies between HwTx-IV(acid) and HwTx-IV(amide) and is likely to be responsible for the potency difference. A C-terminal glycine(acid) may mimic the native carboxamide. Furthermore, a glycine-extended peptide is a common precursor for *in vitro* amidation using PAM [31,32,34]. Consequently, HwTx-IV^G36^(acid) was cloned into the pET-SUMO vector.

### Production and characterisation of HwTx-IV^G36^(acid)

**Figure 6:**
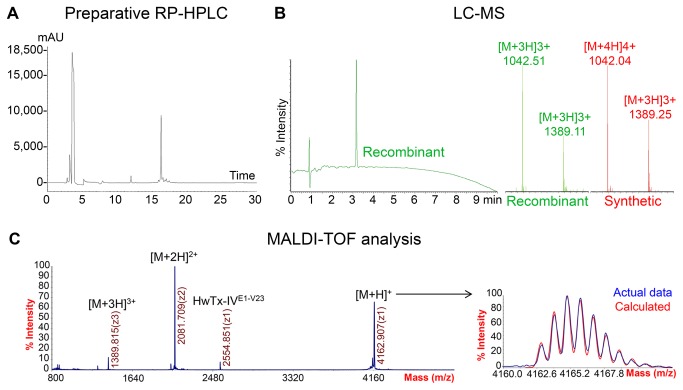
Purification and characterisation of recombinant HwTx-IV^G36^(acid). A) Preparative RP-HPLC. The main peak was collected and lyophilised. B) LC-MS of recombinant and synthetic HwTx-IV^G36^(acid). Synthetic and recombinant HwTx-IV^G36^(acid) are identical. E) MALDI-TOF analysis of recombinant HwTx-IV^G36^(acid). The calculated and observed isotope distributions and m/z ions match. The minor cleaved form HwTx-IV^E1-V23^ was detected again. The theoretical m/z ions are: [M+H]^+^ = 4162.950 m/z, [M+2H]^2+^ = 2081.975 m/z, [M+3H]^3+^ = 1388.317 m/z and [M+4H]^4+^ = 1041.488 m/z.

The glycine extended HwTx-IV(acid) was also expressed in SHuffle T7 Express *lysY* using the pET-SUMO vector, and purified using the same strategy. Following the capture IMAC, proteolytic cleavage and depletion IMAC, the final RP-HPLC polishing step (Figure 6, A) shows that HwTx-IV^G36^(acid) and HwTx-IV(acid) have similar purity. Characterisation by LC-MS (Figure 6, B-D) indicates that purified HwTx-IV^G36^(acid) appears as a single peak and that the corresponding m/z ions are identical to those of the matching oxidised synthetic peptide. MALDI MS analysis (Figure 6, E) demonstrated that the peptide is intact and fully oxidised. A trace of HwTx-IV^E1-V23^ is also detected in HwTx- IV^G36^(acid) final product. Consequently, HwTx-IV(acid) and HwTx-IV^G36^(acid) express and fold equally well in the SUMO/SHuffle, with a slightly lower yield for HwTx-IV^G36^(acid) (Table 1). 

**Table 1:** Amount of recombinant protein produced and yields.

Protein	Fusion protein after Capture IMAC (mg)	Yield (mg/L)	Cleaved peptide after Depletion IMAC (mg)	Yield (mg/L)
HwTx-IV(acid)	8.8	7.4	1.1	0.9
HwTx-IV^G36^(acid)	6.6	5.5	0.8	0.7

The amounts of protein produced were calculated using absorbance at 280nm. The yields refer to the amount of protein produced (mg) per litre of *E. coli* culture.

**Figure 7:**
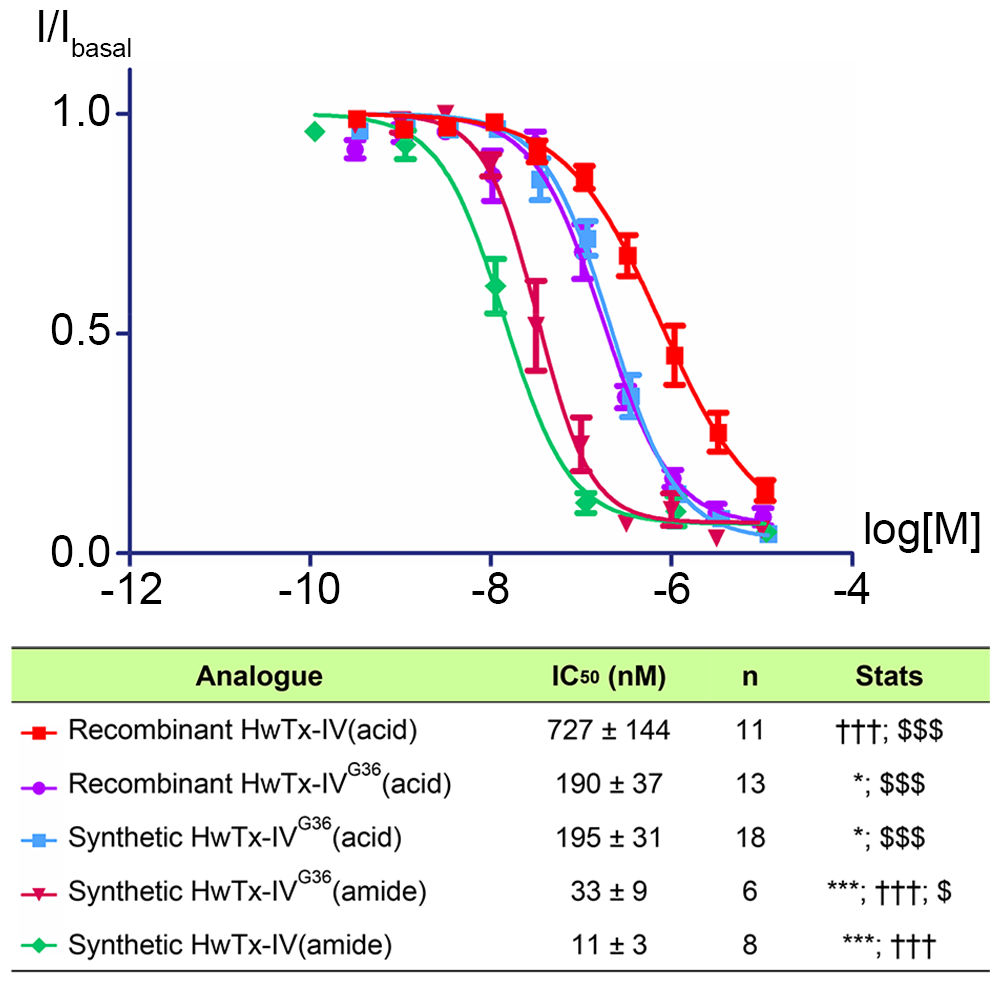
Effect of C-terminal glycine addition on potency. Na_v_1.7 currents were measured from HEK cells stably expressing the hNa_v_1.7 alpha subunit using whole cell voltage clamp. Peak sodium current in the presence of increasing doses of HwTx-IV (10 minutes incubation per dose) is plotted as a fraction of basal current (I/Ibasal). Statistics indicate significant difference in IC_50_ between selected groups (one-way ANOVA with Tukey post-test). * = vs recombinant HwTx-IV(acid), *** = p<0.001; † = vs. synthetic HwTx-IV^G36^(acid), ††† = p<0.001; $ = vs synthetic HwTx-IV(amide), $ = p<0.05, $$$ = p<0.001. No significant difference was observed between the recombinant and synthetic HwTx-IV^G36^(acid).

Electrophysiological characterisation of the synthetic and recombinant HwTx-IV^G36^(acid) shows that both proteins have the same potency with IC_50_ values of 195 ± 31 nM and 190 ± 37 nM, respectively (Figure 7). confirming that they are both correctly folded. In comparison to HwTx-IV(acid), these two proteins are ~3 fold more potent inhibitors of Na_v_1.7, clearly indicating that the potency of HwTx-IV(acid) can be increased by mimicking the C-terminus amide and that processing of the C-terminal glycine to the native amide may not be necessary. Interestingly, glycine extension of the synthetic HwTx-IV(amide) (synthetic HwTx-IV^G36^(amide)), resulted in a 3-fold drop in potency compared to native HwTx-IV(amide).

### Conversion of HwTx-IV^G36^(acid) to HwTx-IV(amide)

PAM-mediated *in vitro* amidation was performed on synthetic HwTx-IV^G36^(acid). The peptide was then analysed by MALDI-MS, and compared to HwTx-IV^G36^(acid) and HwTx-IV(amide) (data not shown). The predominant component after PAM treatment was the starting peptide, with a very minor fraction of the amidated reaction product, although this was not well-resolved. According to Mehta et al., HwTx-IV^G36^(acid) C-terminal Ile35-Gly36 is a relatively good substrate for PAM [32]. The compact structure of HwTx-IV may hinder access to Gly36, and explain why folded HwTx-IV^G36^(acid) appears to be an inappropriate substrate for PAM *in vitro* amidation. Extensive optimisation of the reaction conditions would be required for preparative generation of HwTx-IV(amide) using this strategy.

## Discussion

Discovery of novel toxins from venomous species such as spiders, snakes, frogs, scorpions, sea anemones and cone snails has been widely reported in the last 20-30 years. The evolution-driven optimisation of the active components in venom means that they are a rich source of molecules with unique specificities and properties towards ion-channels and receptors. These peptides can be crucial tools in functional studies and drug development [35]. Whereas identification and isolation of novel peptides is achieved by biochemical procedures, producing them synthetically or recombinantly poses new challenges. The great variety in their sequences and structures means that a single strategy cannot apply to all. 

During the preparation of this manuscript, two reports of ICKs expressed in an *E. coli* strain engineered to enable cytoplasmic disulfide-bond formation were published [11,23]. Pluzhnikov et al. used Origami B to express ω-Lsp-IA as a fusion to thioredoxin in order to increase the yield of folded ICK [11]. Nozach et al. have compared the expression of six ICKs in SHuffle T7 Express *lysY* and Origami B(DE3)pLysS using various fusion partners, including DsbC and thioredoxin [23]. We have similarly used DsbC (and its two thioredoxin domains) to increase the efficiency of cytoplasmic disulfide-bond formation. To the best of our knowledge, we are the first to report the combination of SUMO fusion and SHuffle expression.

SUMO is an advantageous fusion partner for HwTx-IV because it increases solubility of peptides and difficult-to-express proteins [28–30]. Furthermore, it does not contain cysteines which could interfere with the cytoplasmic oxidative folding of HwTx-IV. Thanks to its chaperone activity [27], it may actually enhance folding. High amounts of soluble fusion protein were observed, in comparison to the other fusion systems that we have tried (data not shown), suggesting that SUMO fusion did solubilise HwTx-IV and increased the yield. The SUMO protease cleavage system was also very efficient at producing the native peptide without any residual amino acids at the N-terminus, which is crucial for potency.

Expression in SHuffle generated oxidised His6-SUMO-HwTx-IV(acid), according to non-reducing SDS-PAGE analysis of the IMAC fractions. The presence of multimers indicates that some intermolecular disulfide bridges were also formed in the cytoplasm. Since SUMO does not have cysteines, the bonds had to be between cysteines in HwTx-IV(acid). This would obviously prevent the folding of the native ICK motif. His6-SUMO-HwTX-IV(acid) monomers were purified by fine-tuning the capture IMAC protocol. Polished cleaved HwTx-IV(acid) from this monomeric fraction was very pure, homogeneous, full length folded peptide. It is interesting to note that we did not observe any monomeric His6-SUMO-HwTX-IV(acid) with incorrect disulfide pairing. DsbC and SUMO-assisted oxidation and folding must be responsible for this tendency to form the correct intramolecular bonds within the monomeric fusion proteins.

We compared expression overnight at 16°C to 4 hours at 30°C to look for soluble monomeric fusion protein. As we have established that homogeneous correctly folded HwTx-IV(acid) is produced, it is theoretically possible to further optimise protein expression looking at other parameters such as time of induction and concentration of inducer, co-expression of helper proteins and alternative fusion partners [23,26]. However, this optimisation lies outside the scope of this paper and would only follow a full assessment of structure-function optimisation of HwTx-IV(acid) analogues.

For both recombinant and synthetic approaches, folding or *in vitro* re-folding requires optimisation. Initially, a fraction, if not the majority of the peptide, will have incorrect disulfide-bond pairing and improper folding. The selective elution of monomeric fusion proteins using IMAC offers an easy solution for isolation of correctly folded material. The yield of HwTx-IV(acid) in this system was 0.9mg/L, which is similar to other ICKs expressed in recombinant systems and purified to obtain a homogeneous product [10,11,14,15]. However, the folding propensity of each ICK is different and will affect the total yield. Hence, it is not possible to directly compare the different expression systems unless the same protein is expressed. 

The recombinant and synthetic HwTx-IV(acid) are equally potent on Na_v_1.7 but have ~50 fold drop compared to the native HwTx-IV(amide). HwTx-IV(acid) is however as potent on Na_v_1.7 as HwTx-I (IC_50_ = ~630 nM) which was reported in phase I clinical trial [10]. Structurally, the two toxins are closely related, but where HwTx-I is an unmodified peptide sequence with an acid at the C-terminus, HwTx-IV has a C-terminal amide generated by post-translational modification (conversion of a Glycine-Lysine extended precursor [36]). 

HwTx-IV^G36^(acid) expressed equally well in the SUMO/SHuffle system and showed ~3 fold increased potency on Na_v_1.7 compared to HwTx-IV(acid), with a ~17 fold drop compared to HwTx-IV(amide). The enzymatic conversion of HwTx-IV^G36^(acid) to HwTx-IV(amide) with PAM was very inefficient with only trace amounts of amidated product detectable by MALDI-MS. 

There are relatively few published alternatives to PAM *in vitro* amidation, such as transacylation by Carboxypeptidase-Y of a leucine-extended precursor [33,37], co-expression and secretion of PAM with the protein of interest in a Gram-positive bacteria [38,39], or ammonia cleavage of the thioester generated after DTT induced auto-cleavage of an intein fusion [40]. None of these systems is straightforward and may not be suitable for the compact structure of folded HwTx-IV.

The increased potency of HwTx-IV^G36^(acid), as well as other HwTx-IV(amide) analogues recently characterised in our lab [8], indicates a certain flexibility around the C-terminal sequence. It justifies structure/activity studies around C-terminal acid analogues, where the charge of the carboxylate is neutralised by additional residues or substitutions. Engineering HwTx-IV to achieve a potent carboxylate analogue would be the preferred strategy to obtain a developable Na_v_1.7 inhibitor, since this peptide could be produced recombinantly without the need for C-terminal amidation. 

In summary, we report a novel protocol for recombinant expression of HwTx-IV(acid). It combines several new technologies, and is the first time SUMO has been reported as a fusion partner for an ICK. SHuffle T7 Express *lysY* was used for cytoplasmic expression, and we have optimised the IMAC for selective elution of monomeric fusion proteins. The purified HwTx-IV(acid) was highly homogeneous and correctly folded. This protocol may be applicable to other disulfide rich proteins that have proved difficult to produce in traditional systems. The C-terminal amide of native HwTx-IV is important for potency on Na_v_1.7, but we showed that extending HwTx-IV(acid) with a C-terminal glycine partially restores some lost potency. A structure/activity study of HwTx-IV C-terminus could generate a potent carboxylate analogue which would be ideal for large scale recombinant production.

## Supporting Information

Figure S1 **Electrophysiological properties of hNa_v_1.7.** A) Example whole cell current trace from HEK 293 cells expressing hNa_v_1.7. Voltage was stepped from -120mV to -10mV as indicated by the inset trace resulting in activation of inward hNa_v_1.7 current. Dotted line indicates zero current. B) Current-voltage relationship of hNa_v_1.7. Peak current in response to voltage pulses from -120 to +40mV were normalised to the maximum current response (Imax). Data indicate mean +/- SEM (n = 8).
(TIF)

Figure S2 **Inhibition of hNav1.7 currents by HwTx-IV analogues.** A) Time course of inhibition of hNa_v_1.7 in response to bath application of HwTx-IV(amide) added at increasing concentrations to generate a cumulative dose response. Boxes indicate the time at which each dose of HwTx-IV(amide) was present (in nM). Note that steady state was achieved at each dose within 300s. B-G) Example whole cell current traces taken at steady state in response to varying concentrations of HwTx-IV analogues.
(TIF)
