# Supplementary figures and images for: Recombinant Expression and In Vitro Characterisation of Active Huwentoxin-IV
Source: PLoS One. 2013 Dec 6;8(12):e83202. doi: 10.1371/journal.pone.0083202 (PMC3855799; doi:10.1371/journal.pone.0083202)

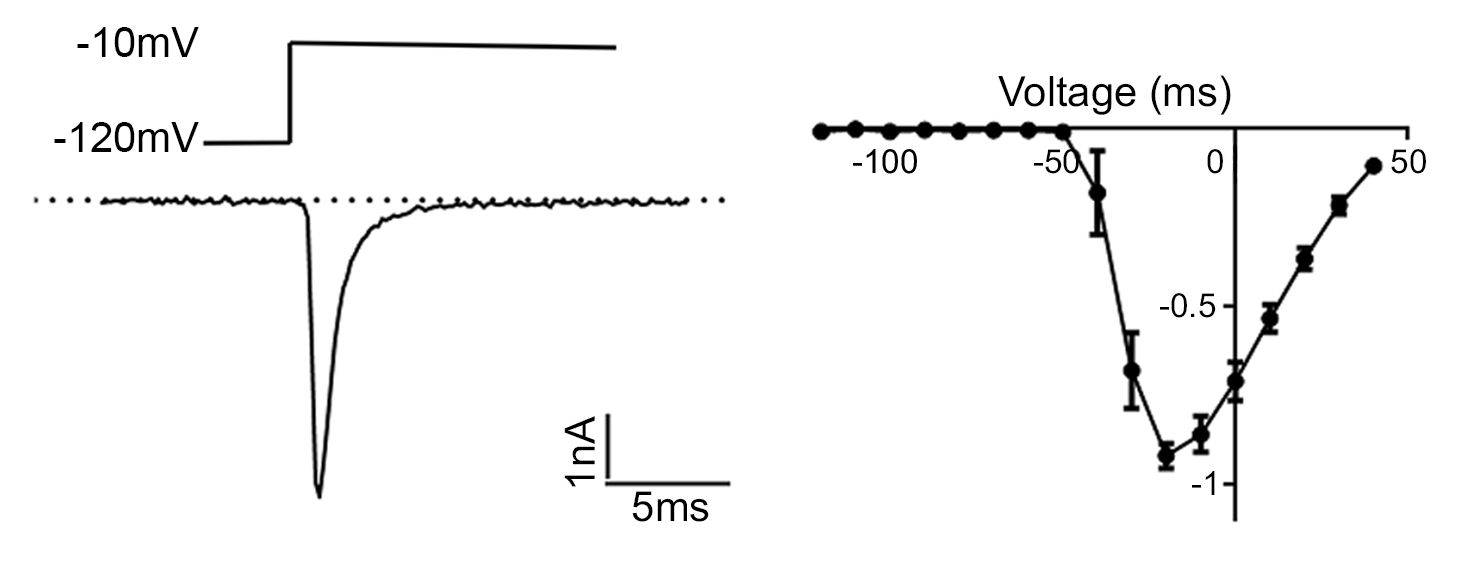

Supplement: Figure S1 — Electrophysiological properties of hNav1.7. A) Example whole cell current trace from HEK 293 cells expressing hNav1.7. Voltage was stepped from -120mV to -10mV as indicated by the inset trace resulting in activation of inward hNav1.7 current. Dotted line indicates zero current. B) Current-voltage relationship of hNav1.7. Peak current in response to voltage pulses from -120 to +40mV were normalised to the maximum current response (Imax). Data indicate mean +/- SEM (n = 8). (TIF) [file pone.0083202.s001.tif]

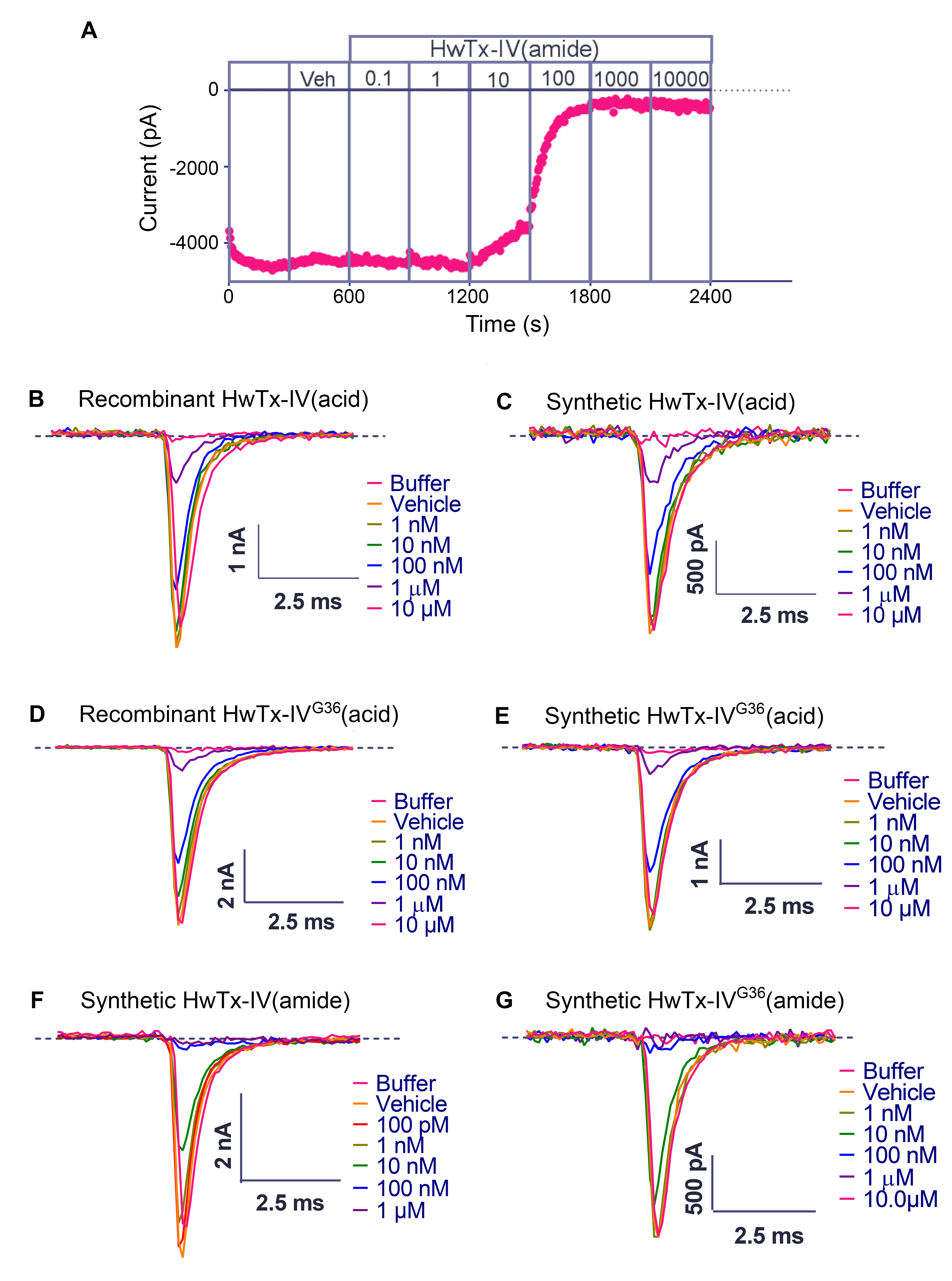

Supplement: Figure S2 — Inhibition of hNav1.7 currents by HwTx-IV analogues. A) Time course of inhibition of hNav1.7 in response to bath application of HwTx-IV(amide) added at increasing concentrations to generate a cumulative dose response. Boxes indicate the time at which each dose of HwTx-IV(amide) was present (in nM). Note that steady state was achieved at each dose within 300s. B-G) Example whole cell current traces taken at steady state in response to varying concentrations of HwTx-IV analogues. (TIF) [file pone.0083202.s002.tif]
